# A Regularization Method for Landslide Thickness Estimation

**DOI:** 10.3390/jimaging10120314

**Published:** 2024-12-10

**Authors:** Lisa Borgatti, Davide Donati, Liwei Hu, Germana Landi, Fabiana Zama

**Affiliations:** 1 Department of Civil, Chemical, Environmental, and Materials Engineering, University of Bologna, 40136 Bologna, Italy; lisa.borgatti@unibo.it (L.B.); davide.donati17@unibo.it (D.D.); 2 Department of Mathematics, University of Bologna, 40126 Bologna, Italy; liwei.hu2@unibo.it (L.H.); germana.landi@unibo.it (G.L.)

**Keywords:** landslide depth estimation, inverse problem regularization, synthetic dataset preparation, balancing principle (BP) for regularization, discretization of mass conservation law, 65M32, 65N22, 86A32

## Abstract

Accurate estimation of landslide depth is essential for practical hazard assessment and risk mitigation. This work addresses the problem of determining landslide depth from satellite-derived elevation data. Using the principle of mass conservation, this problem can be formulated as a linear inverse problem. To solve the inverse problem, we present a regularization approach that computes approximate solutions and regularization parameters using the Balancing Principle. Synthetic data were carefully designed and generated to evaluate the method under controlled conditions, allowing for precise validation of its performance. Through comprehensive testing with this synthetic dataset, we demonstrate the method’s robustness across varying noise levels. When applied to real-world data from the Fels landslide in Alaska, the proposed method proved its practical value in reconstructing landslide thickness patterns. These reconstructions showed good agreement with existing geological interpretations, validating the method’s effectiveness in real-world scenarios.

## 1. Introduction

Landslides represent a significant natural hazard that threaten infrastructure, human lives, and property worldwide [1]. Understanding their behavior and potential impact requires an accurate characterization of their key physical properties, particularly their thickness and volume [2]. This characterization is essential for hazard assessment, risk mitigation, and the design of protective measures [3].

Estimating the morphology of the failure surface of landslides represents one of the most challenging tasks for geoscientists due to the limited availability of subsurface data and, for actively moving landslides, the inability to view the surface that is largely covered by the landslide material. As a result, in most cases, indirect methods are employed to infer the geometry of the basal surface [2], which include graphical reconstructions (based on subsurface data and expert knowledge [4]), simplified geometrical assumptions (considering the basal surface as characterized by circular, elliptic, or log-spiral morphologies [5,6]), and reconstructions based on local surface morphology (e.g., [7,8]). However, these methods often fail to capture the complex three-dimensional nature of landslide bodies and their internal dynamics [9,10]. The behavior of landslide materials adds further complexity as their properties can evolve during movement, affecting both flow dynamics and final deposit geometry [11].

One promising method for estimating landslide depth involves an inverse model based on the mass balance equation coupled with assumptions about the landslide’s rheology [12]. This approach provides a robust framework for depth estimation, especially in scenarios where landslides exhibit complex, non-Newtonian fluid behavior [9,12].

Remote sensing technologies have revolutionized our ability to monitor and analyze landslides at various spatial and temporal scales [13]. In particular, recent advances in Interferometric Synthetic Aperture Radar (InSAR) and Light Detection and Ranging (LiDAR) have created new opportunities for sophisticated analysis of landslide behavior and characteristics [14,15]. These technologies enable precise measurements of surface deformation and displacement patterns [12], providing critical input data for quantitative landslide assessment methods [2].

The problem of determining landslide thickness from surface measurements presents several significant challenges. First, the relationship between surface deformation and subsurface geometry is inherently complex and often poorly constrained. Second, available measurement data typically contain significant noise and uncertainties [16]. Third, the mathematical formulation leads to an ill-posed inverse problem that requires careful numerical treatment for stable solutions.

We present a novel computational framework for estimating landslide thickness from satellite-derived elevation data. At its core, the approach integrates mass conservation principles, leading to the mathematical formulation of an inverse problem, with regularization methods to address the inherent ill-posedness of the problem. This integration of physical modeling with sophisticated numerical techniques allows for robust thickness estimation, even in the presence of significant measurement noise.

The development of our method draws inspiration from regularization techniques based on the Balancing Principle [17], which is widely used in large-scale imaging applications such as image deblurring and tomographic reconstruction [18,19,20]. Regularization techniques are based on the introduction of regularization functions that establish desired characteristics of the solution, and they are weighted by a regularization parameter. For effective results, it is crucial to select appropriate regularization functions based on the specific features of the data and physical model. Moreover, automatic methods for determining the optimal regularization parameter value are essential as this choice critically impacts the quality of the inverse problem solution. Various selection criteria have been proposed in the literature, including the well-known discrepancy principle [21], which requires prior knowledge of noise characteristics; the L-curve criterion, which is based on the relationship between residual norm and regularization terms [22]; and generalized cross-validation approaches that minimize prediction errors [23]. Despite these developments, optimal parameter selection remains an active area of research. A promising recent development is the Balancing Principle (BP), which provides a unified framework for computing both the regularization parameter and the regularized solution. Initially derived from Bayesian statistical analysis for Tikhonov regularization [17], the BP has since been extended to handle multi-parameter regularization problems [24]. Recent studies have demonstrated its effectiveness in imaging applications [25,26], establishing its practical utility across diverse problem domains.

Numerical approaches to inverse problems are usually validated over many test problems. However, no such datasets exist for testing purposes for the application considered in this work. Without test problems, it is impossible to assess the performance of a numerical method for estimating the landslide depth. To fill this gap, we developed a comprehensive synthetic dataset using the Discrete Element Method (DEM) [27]. This dataset provides a controlled environment for testing and benchmarking our method, addressing a significant gap in the field where standardized test cases have been lacking. Studies such as [28,29,30] have typically simulated specific landslide scenarios based on localized data, making them inherently non-reproducible and limiting their broader applicability. By contrast, this study introduced a synthetic model of manageable size to enable efficient, reproducible testing of diverse numerical strategies within a controlled environment.

We further demonstrate the practical utility of our approach through application to the Fels landslide in Alaska [31], showing how the method performs with real-world data.

The main contributions of this work include the following:

- Development of a robust numerical framework for landslide thickness estimation based on mass conservation principles.
- Implementation of a regularization approach based on the Balancing Principle for automatically estimating the landslide thickness and the regularization parameter.
- Creation of a reproducible synthetic landslide dataset for algorithm validation.
- Demonstration of the method’s effectiveness through application to real landslide data [31].

The remainder of this paper is organized as follows. Section 2 presents the mathematical framework, including the problem discretization and regularization approach. Section 3 describes our synthetic landslide dataset and its construction. Section 4 presents numerical results from both synthetic and real-world applications, followed by conclusions and future research directions.

## 2. The Mathematical Framework

This section develops the mathematical framework for estimating landslide thickness, starting from the underlying physical model of mass conservation, presenting the regularization strategy for solving the resulting inverse problem, and then providing details of its numerical implementation.

### 2.1. Physical Model and Mass Conservation

Let the landslide thickness be represented by the function $h:D\times R^{+}\to R$, where D is a closed subset of $R^{2}$. Under the assumption of constant density, the mass conservation yields the following:

(1) $$\frac{\partial h}{\partial t}=-\nabla \cdot \left(\bar{v}h\right),$$

where $\bar{v}\in R^{2}$ is the depth-averaged horizontal velocity of the landslide across the domain D.

Surface velocities can be measured remotely, enabling a simplification under the assumption that, for landslides that are thin relative to their length, the depth-averaged velocity $\bar{v}$ is approximated as $fu_{s}$. Here, $u_{s}\in R^{2}$ denotes the surface horizontal velocity vector (in $m\;s^{-1}$), and f∈(0,1] is a factor dependent on the landslide material’s rheological properties. In landslides governed by a power-law rheological model and a slender flow approximation, the shear strain rate is proportional to the shear stress raised to a given exponent [32]. Specifically, $f=\frac{1}{2}$ corresponds to a linear vertical velocity profile, $f=\frac{2}{3}$ aligns with Newtonian viscous flow, $\frac{2}{3}<f<1$ represents plug flow, and f=1 indicates a rigid sliding block.

Assuming a stable basal surface over the observation period, the rate of change in landslide depth corresponds to the vertical displacement rate, which is denoted by ζ [12]. Thus, the depth evolution model can be expressed as follows:

(2) $$\frac{\partial \zeta }{\partial t}=-\nabla \cdot \left(u_{s}fh\right).$$

In practice, both the vertical displacement rate $\frac{\partial \zeta }{\partial t}$ and the surface velocity $u_{s}$ can be obtained through remote sensing methods such as InSAR (Interferometric Synthetic Aperture Radar) and LiDAR (Laser Imaging Detection and Ranging), which can even image the ground surface through vegetation (see [14] and the references therein).

### 2.2. Discrete Formulation

For numerical treatment, we transformed the continuous model (2) into a discrete system using a finite difference discretization of model (2) on a uniform Cartesian grid with spacing δ. Since the horizontal velocity $u_{s}$ is a bidimensional vector, we can express the continuous depth evolution model as follows:

(3) $$\frac{\partial (h_{f}u_{x})}{\partial x}+\frac{\partial (h_{f}u_{y})}{\partial y}=-\frac{\partial \zeta }{\partial t},$$

where $u_{x}$ and $u_{y}$ denote the components of $u_{s}$ in the *x* and *y* directions, respectively.

To discretize (3), we supposed D as being a rectangular domain in $R^{2}$ and considered a uniform Cartesian grid in D with an equal grid space δ in two coordinates and grid points:

(4) $$\left(x_{i},y_{j}\right),\;i=1,\ldots ,n_{x},\;j=1,\ldots ,n_{y}.$$

By applying the Leibniz product rule, we obtained the following discrete version of Equation (3):

(5) $$\begin{array}{c} {\left(\frac{\partial h_{f}}{\partial x}\right)}_{ij}{\left(u_{x}\right)}_{ij}+{\left(\frac{\partial u_{x}}{\partial x}\right)}_{ij}{\left(h_{f}\right)}_{ij}+{\left(\frac{\partial h_{f}}{\partial y}\right)}_{ij}{\left(u_{y}\right)}_{ij}+{\left(\frac{\partial u_{y}}{\partial y}\right)}_{ij}{\left(h_{f}\right)}_{ij}=-{\left(\frac{\partial \zeta }{\partial t}\right)}_{i,j},\;\;\;\;\;\;\;\;\;\;\;\;\;\;\;\;\\ \;\;\;\;\;\;\;\;\;\;\;\;\;\;\;\;\;\;\;\;\;\;\;\;\;\;\;\;\;\;\;\;\;\;\;\;\;\;\;\;\;\;\;\;\;\;\;\;\;\;\;\;\;\;\;\;\;\;\;\;\;\;\;\;i=1,\ldots ,n_{x},\;j=1,\ldots ,n_{y}. \end{array}$$

We used central finite differences to approximate the first-order partial derivative at the grid points, and we obtained

(6) $$\begin{array}{c} -{\left(u_{x}\right)}_{i,j}{\left(h_{f}\right)}_{i-1,j}+{\left(u_{y}\right)}_{i,j}{\left(h_{f}\right)}_{i,j+1}+\left({\left(u_{x}\right)}_{i+1,j}-{\left(u_{x}\right)}_{i-1,j}+{\left(u_{y}\right)}_{i,j+1}-{\left(u_{y}\right)}_{i,j-1}\right){\left(h_{f}\right)}_{i,j}+\\ -{\left(u_{y}\right)}_{i,j}{\left(h_{f}\right)}_{i,j-1}+{\left(u_{x}\right)}_{i,j}{\left(h_{f}\right)}_{i+1,j}=-2\delta {\left(\frac{\partial \zeta }{\partial t}\right)}_{i,j},\\ \;\;\;\;\;\;\;\;\;\;\;\;\;\;\;\;\;\;\;\;\;\;\;\;\;\;\;\;\;\;\;\;\;\;\;\;\;\;\;\;\;\;\;\;\;\;\;\;\;\;\;\;\;\;\;\;\;\;\;\;\;\;\;\;\;\;\;\;\;\;i=1,\ldots ,n_{x},\;j=1,\ldots ,n_{y}. \end{array}$$

Using the co-lexicographic ordering of the unknowns and zero-padding boundary conditions, the above equations can be written in matrix form as

(7) $$Ah_{f}=b,$$

where the right-hand side $b\in R^{n}$, $n=n_{x}n_{y}$, is determined by the elevation change $\frac{\partial \zeta }{\partial t}$, and the forward operator $A\in R^{n\times n}$ is a penta-diagonal matrix depending only on the surface velocity $u_{s}=\left(u_{x},u_{y}\right)$.

Solving the inverse problem (7) presents the following challenges.

- The coefficient matrix *A* is ill-conditioned and depends on noisy observations $u_{s}$. The right-hand side b is affected by noise due to measurement errors.
- Large-scale data: Typical datasets cover several square kilometers at a resolution ranging from 1 to 10 m, leading to large-scale problems.
- Difficulties in evaluating the performance of numerical methods due to the lack of readily available synthetic data and the absence of a simple, standardized computational tool.

To address these issues, we propose an automatic regularization method, detailed in the following paragraph, coupled with a synthetic dataset for validation, as described in Section 3.

### 2.3. The Regularization Method

In the inverse problems community, regularization methods are applied to tackle linear ill-posed problems. In particular, variational regularization methods involve solving the following minimization problem:

(8) $$\min_{h_{f}\geq 0}{∥Ah_{f}-b∥}^{2}+\lambda \psi \left(h_{f}\right),$$

where ψ is the regularization function, and λ>0 is the regularization parameter realizing the trade-off between the data fidelity, which is represented by the least square distance to the data b and the solution smoothness, which is expressed by $\psi (h_{f})$. Here and henceforth ∥·∥ denotes the L2 norm. Through using the BP, we determined the regularization parameter $\lambda^{*}$ and the corresponding regularized scaled depth ${h_{f}}^{*}$ as the solution of the non-linear system:

{(9a)hf*=argminhf≥0∥Ahf−b∥2+λ*ψ(hf),(9b)λ*=∥Ahf*−b∥2γψ(hf*),

with

(10) $$\psi \left(h_{f}\right)={∥Lh_{f}∥}^{2}+\varepsilon ,$$

where $L\in R^{n\times n}$ is the discrete Laplacian operator, ε is a positive threshold preventing division by zero, and γ is a positive parameter. It is evident that the BP determines the optimal regularization parameter $\lambda^{*}$ by balancing, up to the multiplicative parameter γ, the data-fidelity term with the regularization function ψ. We used the fixed-point method proposed in [17] to determine a solution $\left(h_{f}^{*},\lambda^{*}\right)$ to the nonlinear system (9a)-(9b). Starting from an initial guess $\lambda^{\left(0\right)}$, at each *k*th iteration, this fixed-point method computes an approximation ${h_{f}}^{\left(k\right)}$ as the solution to the constrained minimization problem

(11) $${h_{f}}^{\left(k\right)}=\arg\min_{h_{f}\geq 0}\;∥Ah_{f}{-b∥}^{2}+\lambda^{\left(k\right)}\psi \left(h_{f}\right).$$

Then, a new estimate $\lambda^{\left(k+1\right)}$ of the regularization parameter was obtained as

(12) $$\lambda^{\left(k+1\right)}=\frac{∥A{h_{f}}^{\left(k\right)}{-b∥}^{2}}{\gamma \psi ({h_{f}}^{\left(k\right)})}.$$

The fixed-point method is sketched in Algorithm 1.

At each iteration of Algorithm 1, the computation of the iterate ${h_{f}}^{\left(k\right)}$ requires the solution of the non-negatively constrained least-squares problem (11). To this aim, we used the Gradient Projection (GP) method with Barzilai and Borwein rules for the step length selection [33,34].

In our implementation, we stopped Algorithm 1 when the relative distance between the two successive approximations of the regularization parameter became less than a given tolerance Tol∈(0,1), i.e., when

(13) $$\frac{|\lambda^{\left(k+1\right)}-\lambda^{\left(k\right)}|}{\lambda^{\left(k+1\right)}}\leq Tol,$$

or when a maximum number $k_{\max}$ of iterations was reached.

Convergence of the fixed-point scheme to a solution of the nonlinear system (9a)-(9b) was proven for the general case of several regularization terms in [26]. The convergence analysis was also applied to a single regularization term in (9a)-(9b).
**Algorithm 1** Fixed-point method for landslide thickness estimation.    **Input**    A: Forward operator matrix    b: Right-hand side vector (elevation change data)    Tol: Tolerance for convergence    $\;\;k_{\mathrm{GP}}$: Maximum GP iterations    γ: Balancing parameter    ε: Threshold value in (10)    **Output**    $\;\;{h_{f}}^{*}$: Estimated landslide thickness   $\;\;\lambda^{*}$: Optimal regularization parameter  1:// Initialization    2:Compute initial guess ${h_{f}}^{\left(0\right)}$ using early-stopped GP on $\min_{h_{f}\geq 0}{∥Ah_{f}-b∥}^{2}$    3:$\lambda^{\left(0\right)}=\frac{∥A{h_{f}}^{\left(0\right)}{-b∥}^{2}}{\gamma \psi ({h_{f}}^{\left(0\right)})}$ {Initial regularization parameter}  4:k=0 {Iteration counter}  5:**repeat**   6:   k=k+1    7:   // Solve regularized minimization problem using GP method    8:   ${h_{f}}^{\left(k\right)}=\arg\min_{h_{f}\geq 0}\{∥Ah_{f}-b{∥}^{2}+\lambda^{\left(k-1\right)}\psi \left(h_{f}\right)\}$ $\psi \left(h_{f}\right)={∥Lh_{f}∥}^{2}+\epsilon$, L is Laplacian}  9:   // Update regularization parameter  10:   $\lambda^{\left(k\right)}=\frac{∥A{h_{f}}^{\left(k\right)}{-b∥}^{2}}{\gamma \psi ({h_{f}}^{\left(k\right)})}$  11:**until** $\frac{|\lambda^{\left(k\right)}-\lambda^{\left(k-1\right)}|}{\lambda^{\left(k\right)}}\leq Tol$ OR $k\geq k_{\max}$  
12:**return** ${h_{f}}^{*}={h_{f}}^{\left(k\right)}$, $\lambda^{*}=\lambda^{\left(k\right)}$

## 3. The Synthetic Landslide Data

To test numerical methods, it is necessary to have ground truths that allow us to assess the quality of the reconstructions. However, creating ground-truth basal surfaces from large, active landslides requires the use of field and subsurface data that are complex and costly to collect. It represents one of the main challenges in applied geology and landslide hazard management [31], particularly for landslides characterized by challenging terrain (e.g., due to rockfall risk or surface cracking) or those that are located in inaccessible areas [16]. Therefore, we developed a realistic synthetic landslide model that provides complete control over slope morphology, basal surface, and material properties, allowing for precise testing of the proposed reconstruction method. We provide a brief explanation in this paragraph, with the technical details deferred to in Appendix A.

**Figure 1:**
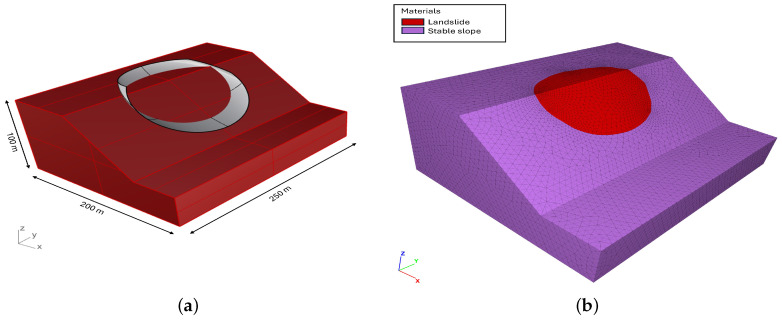
Overview of the synthetic landslide model: (**a**) three-dimensional geometry created in Rhinoceros; and (**b**) synthetic landslide model geometry in 3DEC, showing the different materials that form the model slope.

**Figure 2:**
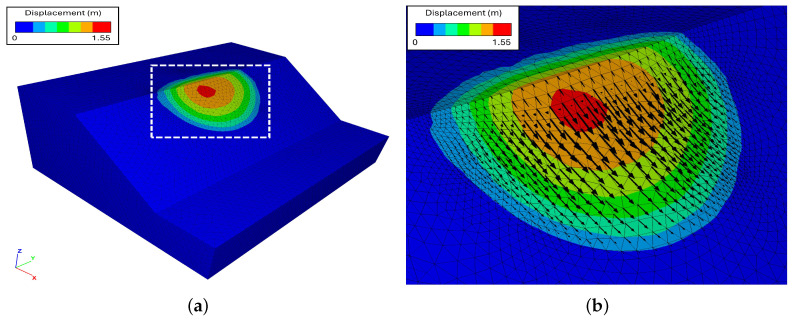
Overview of the synthetic landslide model: (**a**) displacement magnitude distribution at the end of the simulation ($t_{1}$), where the white square outlines the area depicted in (**b**); and (**b**) detail of the landslide area, with the displacement vectors displayed as black arrows.

**Figure 3:**
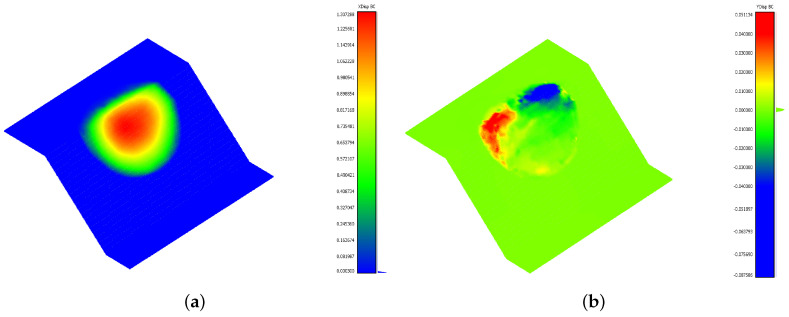
Data of a synthetic landslide: (**a**) x-displacement component; (**b**) y-displacement component.

**Figure 4:**
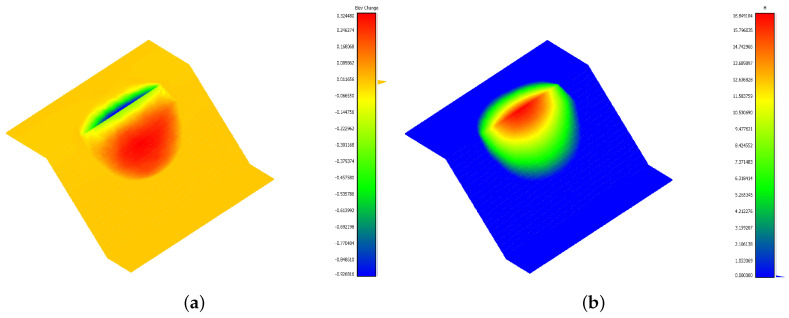
Data of a synthetic landslide: (**a**) elevation change; (**b**) landslide thickness.

The model, representing a simple rotational landslide, was created in the CAD software Rhinoceros [35] (Figure 1a), and it was meshed with tetrahedral elements using the Griddle plugin [36]. Simulations were conducted using the Discrete Element Method (DEM) in 3DEC [37], where the materials assigned to the synthetic landslide and stable slope had distinct mechanical properties (Figure 1b). Custom FISH and Python scripts were employed to extract the model output data at the initial $t_{0}$ and final $t_{1}$ times, including the displacement and elevation change on a user-defined rectangular grid of size 160×200 (Figure 2a,b). This data transformation ensures compatibility with the basal surface reconstruction process and provides the displacement components *x* and *y* reported in Figure 3. The elevation change b and landslide thickness $h_{f}$, which are represented in Figure 4, allow for the accurate evaluation of the reconstruction quality based on the known model geometry.

## 4. Numerical Results

The numerical experiments in this section illustrate the performance of Algorithm 1. In Section 4.2, we validate the algorithm using the synthetic data described in the previous section, allowing us to assess the quality of the computed results by comparing them with a reference value. Then, in Section 4.3, we show the application of Algorithm 1 on a real dataset. We begin by describing the experimental setting in the following paragraph.

### 4.1. Experimental Setting

All experiments were conducted using MATLAB R2023b on an Apple M1 computer with 16 GB of RAM. In our experiments, the initial regularization parameter $\lambda^{\left(0\right)}$ was computed as follows:

(14) $$\lambda^{\left(0\right)}=\frac{∥A{h_{f}}^{\left(0\right)}{-b∥}^{2}}{\psi ({h_{f}}^{\left(0\right)})},$$

where ${h_{f}}^{\left(0\right)}$ is obtained by early-stopped GP iterations applied to the least-squares problem

(15) $$\min_{h_{f}\geq 0}\;{∥A{h_{f}}^{\left(0\right)}-b∥}^{2}.$$

In Algorithm 1, the tolerance Tol for the relative distance between two successive parameter approximations (13) was fixed as equal to 0.1, and a maximum of $k_{\max}=10$ iterations was allowed. The GP iterations at Step 3 were arrested when the relative distance between the two iterates became less than the tolerance $Tol_{g}p=10^{-7}$ or after $k_{g}p=5\times 10^{6}$ iterations. We highlight that an accurate solution of the minimization problem (11) is essential to ensure the convergence of the fixed-point method implemented by Algorithm 1. The values $\varepsilon =\frac{1}{2}10^{-10}$ and γ=3 were fixed as they were suitable for synthetic and real datasets.

### 4.2. Results on the Synthetic Landslide Data

**Figure 5:**
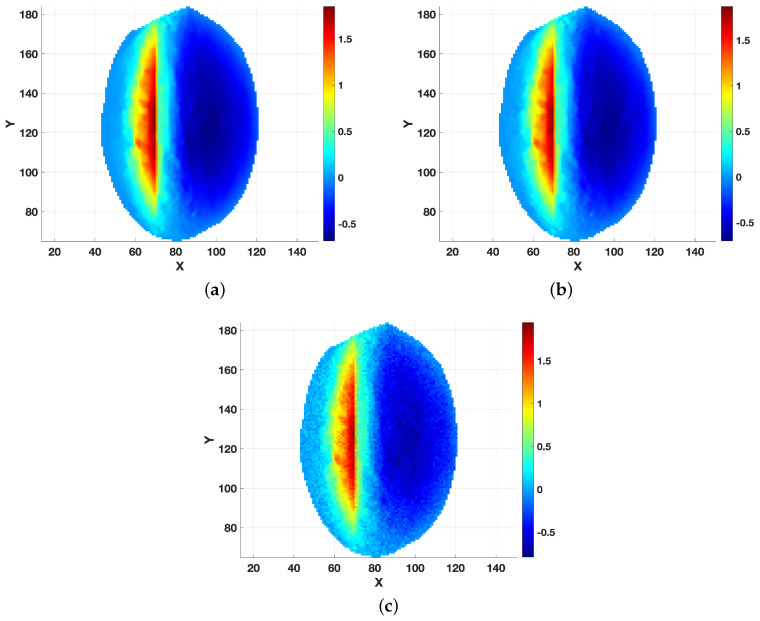
Elevation r.h.s b. (**a**) Noiseless r.h.s; (**b**) noisy r.h.s with a noise level σ=0.01; and (**c**) noisy r.h.s with a noise level σ=0.05.

For the numerical tests, we used artificially generated ground-truth data for the landslide thickness ${h_{f}}^{*}$ and surface velocities $\left(v_{x}^{*},v_{y}^{*}\right)$ obtained, as reported in Section 3. The synthetic dataset represents a landslide over a domain of $160\times 200\;m^{2}$. To simulate realistic conditions, Gaussian noise was added to the data, yielding noisy r.h.s. $b=A{h_{f}}^{*}+\sigma w$, with ∥w∥=1 and a noise level σ in the range $\left[10^{-4},10^{-1}\right]$. The effect of such noise on the data is visualized in Figure 5 for the cases σ=0.01,0.05.

Table 1 summarizes the results of the synthetic data experiments for varying noise levels from σ=0.1 to $10^{-4}$. The first column shows the noise level σ applied to the synthetic data. The second column presents the relative error $\frac{∥h_{f}-h_{f}^{*}∥}{∥h_{f}^{*}∥}$, measuring the accuracy of our reconstruction compared to the ground truth. The third column shows the final regularization parameter λ, which was computed in the last iteration of Algorithm 1. The fourth column reports the number of fixed-point iterations *k* needed to reach convergence. The fifth column displays the total number of GP iterations $k_{GP}$ performed across all fixed-point iterations. The sixth column gives the total computation time in seconds.

The results show that, as the noise decreased from σ=0.1 to σ=0.0001, the relative error reduced from $6.233\times 10^{-2}$ to $9.877\times 10^{-3}$, while the final regularization parameter λ decreased from 7.912 to $2.772\times 10^{-4}$. The number of fixed-point iterations ranged from 8 to 19, with the most iterations required at σ=0.05. The computational time remained relatively stable across all noise levels, averaging around 2 s.

These results demonstrate that the proposed method maintains stability and accuracy across a wide range of noise conditions while retaining reasonable computational efficiency. The automatic parameter selection through the Balancing Principle effectively adapts the parameter value to different noise levels, as evidenced by the systematic variation in λ values.

**Figure 6:**
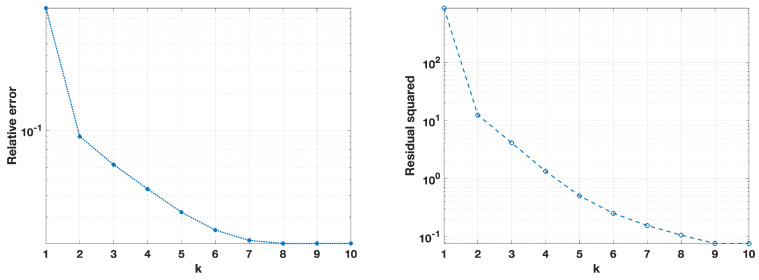
Noise level σ=0.01, relative error on the (**left**), and squared residual norm on the (**right**) for each iteration *k*.

**Figure 7:**
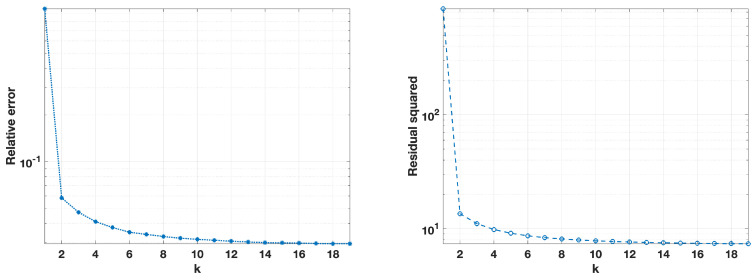
Noise level σ=0.05, relative error on the (**left**), and squared residual norm on the (**right**) for each iteration *k*.

**Figure 8:**
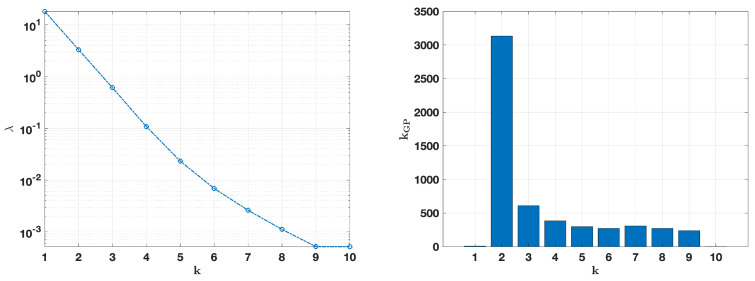
Noise level σ=0.01. On the (**left**): computed values of the regularization parameter $\lambda^{\left(k\right)}$. On the (**right**): the number of internal iterations of the GP method at each iteration *k*.

**Figure 9:**
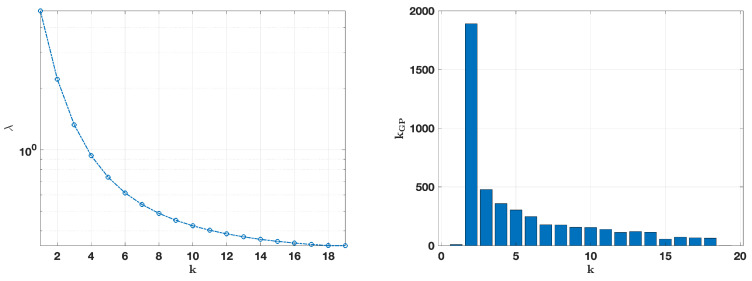
Noise level σ=0.05. On the (**left**): computed values of the regularization parameter $\lambda^{\left(k\right)}$. On the (**right**): the number of internal iterations of the GP method at each iteration *k*.

Figure 6, Figure 7, Figure 8 and Figure 9 provide a visual analysis of the algorithm performance for two representative cases relative to the intermediate noise levels σ=0.01,0.05, where the focus was on critical metrics for each update of the regularization parameter, as defined by the fixed-point iterations. Figure 6 and Figure 7 depict the convergence trends of the relative error and squared residual norm over the fixed-point iteration *k*. On the left side, the relative error decreased rapidly as *k* increased. The squared residual is displayed on the right side, and it similarly decreased across iterations, indicating that the algorithm was effectively refining the solution with each update.

Figure 8 and Figure 9, on the left, show the evolution of the regularization parameter λ across *k* iterations, with λ decreasing as the algorithm progressed, which supported solution stability by gradually reducing the regularization strength. The number of internal iterations for the GP method corresponding to each *k* iteration is shown on the right. In both cases, the GP iterations were the highest at the initial fixed-point steps and diminished over time, indicating reduced computational demand as the solution stabilized.

**Figure 10:**
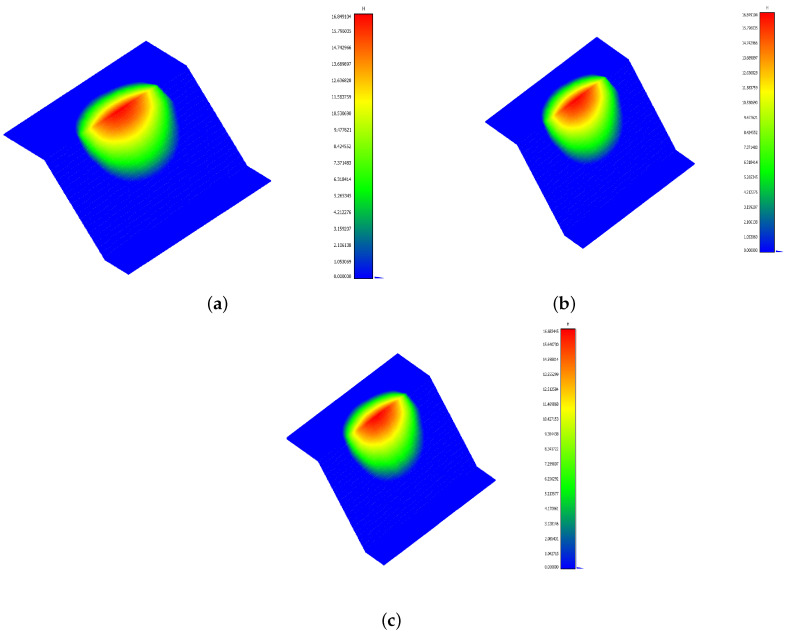
(**a**) Ground truth thickness. (**b**) Computed thickness with a noise level σ=0.01. (**c**) Computed thickness with a noise level σ=0.05.

**Figure 11:**
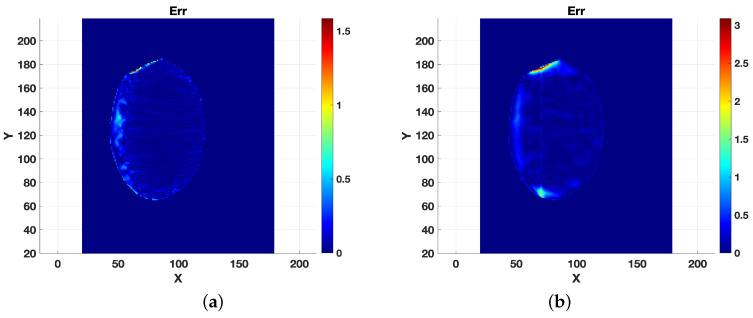
Error maps. (**a**) Noise level σ=0.01, (**b**) noise level σ=0.05.

The accuracy of the computed thickness $h_{f}$ is shown in Figure 10 and Figure 11, which display $h_{f}$ and the absolute error values at each grid point. In Figure 11, we can observe that the most significant errors were concentrated near the boundaries. At the same time, the central region of the sliding surface was accurately reproduced, as highlighted in Figure 10, indicating that the algorithm effectively captured the core structure of the sliding surface, with minimal error in the interior even as slight discrepancies appeared along the edges.

### 4.3. Analysis of a Real Dataset

**Figure 12:**
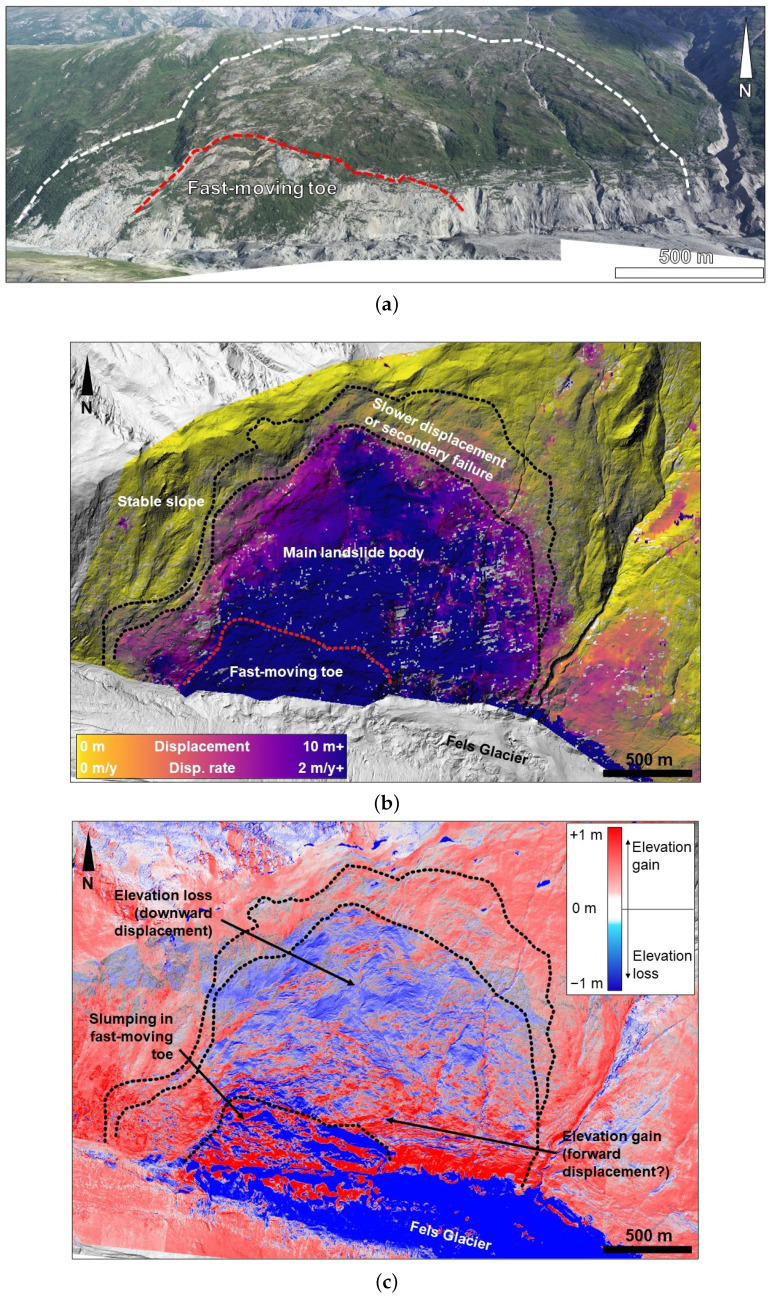
Overview of the Fels landslide and its displacement: (**a**) View of the Fels landslide from the opposite slope; (**b**) displacement magnitude map derived from the SAR analysis described in [31]; and (**c**) the elevation change that occurred between 2014 and 2016, as derived from repeated airborne LiDAR data [15].

In the previous section, the proposed approach was tested using a synthetic dataset characterized by a known geometry and controlled material parameters. Such conditions, however, are seldom met (if ever) in common practice due to the inherent complexity of natural systems (e.g., the spatial variability of material characteristics, geological heterogeneity, etc.), which introduce significant uncertainties and noise in the input data [16]. In this section, the numerical approach is tested using real landslide data. The slope selected for the analysis is the Fels landslide, a large, extremely slowly moving landslide located in central Alaska (US). The landslide is located within the Alaska Range, specifically on the northern slope of Fels Glacier valley, a tributary of the Delta River valley [31]. The unstable slope dips 20–$30^{\circ }$ to the south, and its steepness increases to 40–$50^{\circ }$ nearing the glacier that partly occupies the valley floor. The landslide area is about $2.3\;{\mathrm{km}}^{2}$, and it extends 1400m in the E-W direction and 1600m in the N-S direction between elevations of 1490m and 920m above sea level (a.s.l., Figure 12a). This slope instability has been known since 2013 [38], and, from 2010, it has been monitored using various remote sensing methods, including airborne LiDAR and SAR [31]. In particular, SAR data was used to quantify the displacement in E-W, N-S, and up-down directions, which allowed the magnitude, direction, and plunge of the displacement vectors to be computed (Figure 12b, [15]). SAR monitoring was set to investigate two 5-year-long time windows, namely 2010–2015 and 2015–2020. The computed displacements are displayed using raster maps with a 1m resolution. However, the raw data derived from the SAR analysis has a resolution of about 47 by 51m in the N-S and E-W directions, respectively. SAR analyses showed that the displacement rates spatially varied across the landslide area. In the lower slope, a wedge-shaped block displaced at significantly greater rates (in excess of 5 m/a) than the rest of the landslide. Such a block is referred to as a “fast moving toe” [15], and it displayed signs of rotational movement (i.e., displacement along a circular basal surface) as opposed to the rest of the landslide where translational deformation (i.e., along a planar surface) was inferred [31]. The change in elevation across the entire slope area was measured by means of repeated LiDAR surveys, which were conducted in 2014 and 2016, providing a “change detection” raster map with a 1m resolution (Figure 12c). The results show an overall elevation loss in the upper part of the slope (indicating a predominantly vertical displacement) and an elevation gain in the central part (associated with predominantly horizontal deformation). In the fast-moving toe, the LiDAR change detection map showed bands of elevation gain and loss, indicating the shearing and separation of this volume into multiple blocks [31]. From the datasets, it was possible to derive the input data for Algorithm 1. The *x*- and *y*-components of the horizontal displacement were derived from the SAR map for the 2015–2020 time window, whereas the values of the elevation change were derived from the ALS change detection analysis. Temporally averaged values were considered for the datasets to account for the displacement over one year, preliminarily assuming constant rates for the observed displacements during the monitoring period.

**Figure 13:**
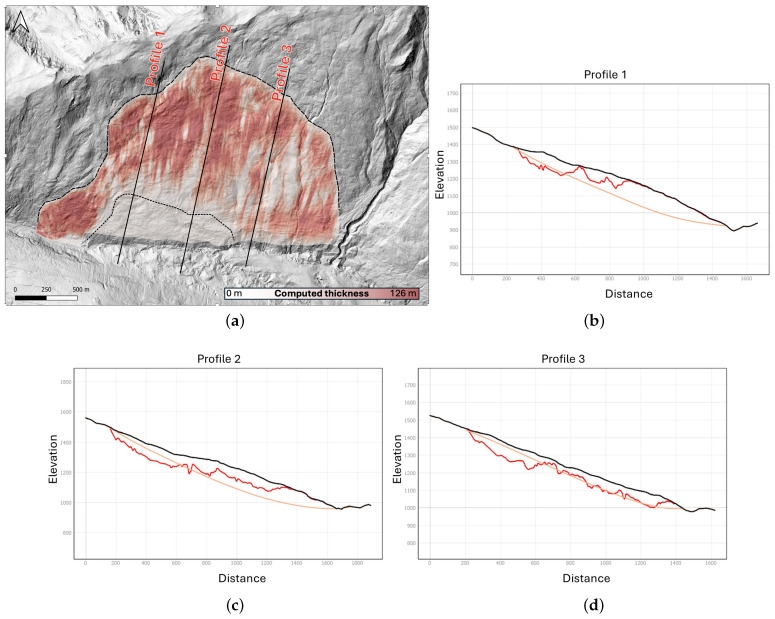
Overview of the computed thickness map: (**a**) The computed thickness map (shades of red) and location of the profiles within the landslide area. The basemap is a hill-shaded relief map derived from the 2016 LiDAR dataset. Section lines are indicated in solid black. Landslide boundaries are marked by the dashed line, while the dotted black line shows the boundary of the fast moving toe. (**b**–**d**) Profiles 1–3 show the morphology of the basal surface inferred with our method and the VIM method. The horizontal axis represents the distance from the upper part of the slide. The vertical axis holds the value of the surface elevation (black), the elevation of the basal surface inferred with VIM (yellow), and our proposed method (red), respectively.

The thickness computed using Algorithm 1 (Figure 13a) was then subtracted from the ground elevation map, obtaining a map of the basal surface elevation. Using the geographic information software QGIS [39], two-dimensional sections were extracted, showing the morphology of the basal surface with respect to the ground elevation. Then, the reconstructed surface was compared with the results obtained from a VIM reconstruction that was manually performed (Figure 13c). The VIM reconstruction was inherently characterized by uncertainties (related to the noise in the SAR dataset) and some degree of subjectivity; however, considering the geological characterization and interpretation described in previous studies [31], the results are geologically sound. The application of Algorithm 1 to the Fels landslide data shows promising results. The surface appears to follow roughly the VIM reconstruction. The computed thickness appears to exceed the VIM data in the upper part of the slope (above 1250 m a.s.l.), and it is significantly lower in the area of the fast-moving toe.

## 5. Conclusions

The aim of this work was to develop a numerical framework for determining the thickness of landslide movements based on the mass conservation approach within the context of linear inverse problems. Synthetic data were generated to perform tests in a controlled environment. Starting from a mass-conservation model discretized with finite differences, an automatic regularization method based on the Balancing Principle was implemented, yielding good results with the synthetic data. This method was then applied to real landslide data, obtaining encouraging preliminary results. The key advantages of our method include the following: (1) automatic parameter selection through the Balancing Principle, eliminating the need for manual tuning; (2) robust performance across different noise levels, as demonstrated by relative errors reducing from $6.233\times 10^{-2}$ to $9.877\times 10^{-3}$ as the noise decreased from σ=0.1 to σ=0.0001; and (3) computational efficiency, with consistent processing times around 2 s. Through our experiments, we verified two key hypotheses: first, that mass conservation principles can effectively reconstruct landslide thickness from surface measurements when coupled with appropriate regularization techniques; and, second, that the Balancing Principle can automatically determine optimal regularization parameters and regularized thickness for this geophysical application. The method’s effectiveness was validated on both synthetic data and the real-world Fels landslide case, where reconstructed basal surfaces aligned well with existing geological interpretations.

However, some challenges related to applying the proposed method to analyze real datasets were noted. Compared to synthetic data, datasets derived from real case studies are characterized by a significant amount of noise, which can be caused by local geological conditions and the technologies and methods employed for collecting displacement data. Landslides or instabilities that affect the slope surficial layer and/or the presence of multiple sliding surfaces can result in significant spatial variation in the displacement rates across the landslide area [16]. In such cases, the complex distribution of the measured surface deformation cannot be easily reconducted to a specific and unique basal surface. The technology and method used for measuring the displacement also represents a source of noise in the input dataset. In the synthetic landslide analysis, each input point feature is associated with the precise measurement of surface elevation change and horizontal displacement at the exact sampling location. In contrast, when real sites are investigated, displacement rates and distribution are commonly displayed through raster maps, where the size of the pixel depends on the characteristics of the sensor and the processing method and the value of the displacement is averaged within the area of the pixel itself. Moreover, each survey method is inherently characterized by varying precision and accuracy, which, in turn, will affect the amount of noise that occurs in the dataset.

To mitigate the sensitivity to data noise, in addition to preliminary denoising filtering, alternative discretizations of the mass balance equation could be explored. Moreover, to improve algorithm accuracy on the fast-moving toe, future investigations will include developing regularization methods that adapt the value of the regularization parameters to the velocity of the landslide zones.

## Figures and Tables

**Table 1:** Results of the synthetic data experiments for varying noise levels.

Noise	Relative Error	Final Parameter	Fixed-Point Iterations	Total GP Iterations	Time
σ	$\frac{\left|\right|h_{f}-{h_{f}}^{*}\left|\right|}{\left|\right|{h_{f}}^{*}\left|\right|}$	λ	k	$k_{\mathrm{GP}}$	s
0.1	$6.233\times 10^{-2}$	7.912	11	3422	1.17
0.07	$4.694\times 10^{-2}$	2.087	9	2557	0.96
0.05	$2.954\times 10^{-2}$	$3.385\times 10^{-1}$	19	4814	1.63
0.01	$1.214\times 10^{-2}$	$5.122\times 10^{-4}$	10	5048	1.79
0.001	$1.054\times 10^{-2}$	$2.912\times 10^{-4}$	8	5026	1.80
0.0001	$9.877\times 10^{-3}$	$2.772\times 10^{-4}$	8	5334	1.74

## Data Availability

Dataset available on request from the authors.

## Appendix A. Description of the Synthetic Data

To test the numerical method presented in this paper, we defined a synthetic landslide model that provides full control over the morphology of the slope and the landslide basal surface, the boundary conditions, and the mechanical characteristics of the materials. In this way, we can evaluate the quality of the reconstructed basal surface based on the prior knowledge of the model geometry. In this analysis, the three-dimensional geometry of a simple rotational landslide (which was characterized by a circular or ellipsoidal basal surface) [40] was created using the CAD software Rhinoceros [35] (Figure 1a), and it was successively meshed with tetrahedral elements using the Griddle plugin [36].

The progressive deformation and displacement of the synthetic landslide were then simulated using the distinct element method DEM [41], which was implemented in the commercial software 3DEC [37]. By exploiting the distinct element method, 3DEC allows stress, deformation, and failure within a three-dimensional geometry (e.g., of a slope or a tunnel) to be computed. It also allows model data (e.g., deformation at nodes and within tetrahedral zones) to be extracted with built-in commands or custom scripts (which were created using a software-specific programming language called FISH). Figure 1b shows the deformation of the synthetic landslide model computed in 3DEC.

The synthetic landslide model used in this analysis was constituted by two different materials, which were assigned to the landslide and to the stable part of the slope, respectively (Figure 1b). The landslide material was characterized by weaker mechanical parameters compared to the stable slope, as reported in Table A1, which allowed the landslide volume to deform and displace relatively with the rest of the slope.

**Table A1:** Mechanical parameters of the simulation materials.

Material	Constitutive Model	Cohesion	Friction Angle
Landslide	Mohr–Coulomb	0.5 kPa	25°
Stable slope	Mohr–Coulomb	8 kPa	35°

A combination of custom FISH and Python scripts was developed to extract, from the synthetic landslide model, the input data required for the basal surface reconstruction (i.e., the elevation change and horizontal displacement components along the user-defined rectangular grid). The FISH script allowed for the ID number of all the triangular faces forming the surface of the model to be exported in table format as a .csv file, hereafter referred to as “model output table”, together with the coordinates of the vertex gridpoints of the simulation at times $t_{0}$ and $t_{1}$ (Figure 2a).

Such a dataset cannot be used directly as the input for the basal surface reconstruction because the model gridpoints are not organized in a rectangular grid but are randomly located. Thus, a separate script was created in Python, which allows for the *x*- and *y*-component of the horizontal displacement at all points of a user-defined rectangular grid to be computed. To do so, for each point of the grid, the ID of the enclosing triangular face (at $t_{0}$) is derived from the model output table. The Cartesian coordinates of the point were then converted to barycentric coordinates within the triangular face, allowing the position of the point inside the triangle to be tracked from $t_{0}$ to $t_{1}$, even as the triangle was deformed. The conversion from barycentric coordinates back to Cartesian coordinates allowed the *x*- and *y*-component of the displacement vector to be accurately computed for each point of the rectangular grid.

To derive the vertical change, i.e., b in (2), the coordinate in the *z* of each point was obtained by interpolating the surface of the enclosing triangular face at $t_{0}$ and $t_{1}$ and then computing the difference. Using a similar approach, the model’s basal surface elevation was also computed for each point of the rectangular grid considering the material boundary as the landslide base. By subtracting the basal surface elevation from the surface elevation at $t_{0}$, the ground-truth thickness of the landslide ($h_{f}$ in (2)) was computed.

The dataset derived from this computation was finally exported in a table format and includes, for each point, the following: the *x* and *y* coordinates, the elevation change (i.e., the change in ζ between $t_{0}$ and $t_{1}$), the *x*- and *y*-component of displacement, and the initial landslide thickness.
